# Rebuilding resilient health systems for Europe

**DOI:** 10.1016/j.lanepe.2021.100238

**Published:** 2021-10-07

The COVID-19 pandemic has created a unique opportunity to re-evaluate and reimagine health system governance and structures, to be better prepared for future catastrophic events, such as a pandemic, extreme weather events, or economic crises. Against this backdrop, *The Lancet Regional Health – Europe* presents a Series of 11 papers on rebuilding resilient health systems for Europe, addressing the lessons that have been learnt during the pandemic, changes that have been adopted, and the transformations that will be needed to achieve health system resilience—the ability to prepare for, manage (absorb, adapt, and transform), and learn from shocks.

During global health emergencies, coordination between countries is of prime importance to achieve better mitigation outcomes domestically and internationally. Mark Jit and colleagues explore how multilateral collaboration between countries, in particular collaboration around issues with economic consequences, is crucial for successful responses to public health emergencies linked to infectious disease outbreaks. The importance of multilateralism is underscored by the estimates of the International Chamber of Commerce Research Foundation—should countries continue to pursue an uncoordinated approach to vaccine distribution, the world risks global gross domestic product losses of as much as US$ 9·2 trillion.

Collaboration should also be extended beyond the usual infrastructures and disciplines towards global early-warning surveillance at the human, animal, and environmental interface, as a key pillar of preparedness. Marion Koopmans and colleagues outline their perspectives on future development of global genomic surveillance strategies and highlight the importance of metagenomic approaches within the framework of the One Health approach to provide a true early warning system. It is clear that novel data streams have the potential to reshape epidemic preparedness and response, but there remains a pressing need to address issues around data sharing, sustainability, and scalability that could hinder future epidemic preparedness, as discussed by Adam J Kucharski and colleagues.

Regionally, the resilience of European medicine regulators has never been tested to the extent as in the pandemic, due to the unprecedented need to evaluate and approve new therapeutic and preventive medicines, while maintaining their supervisory roles for all other medicines in the EU. Marco Cavaleri and colleagues provide a concise perspective on what has been happening at the European Medicines Agency (EMA) in relation to the pandemic and some of the early lessons that will help reshape medicines regulation in the post-COVID-19 era and extend the EMA's mandate, reinforcing its role in crisis preparedness and response.

A welcome step has been the establishment of the EU Health Emergency Preparedness and Response Authority (HERA)—envisaged to play a similar role to the US Biomedical Advanced Research and Development Authority (BARDA)—that could enable a more coordinated and comprehensive European response to future major threats to health. Michael Anderson and colleagues navigate the role of HERA in coordinating responses to major health threats in Europe and beyond. HERA will also be expected to have a global focus that is aligned with the EU commitment to ensure fair and equitable access to medical countermeasures in low-income and middle-income countries (LMICs). This is particularly important in light of the findings from Katrina Perehudoff and colleagues, who present evidence of the effects of EU law, regulation, and policy on access to medicines in non-EU LMICs. Their study illustrates that EU policy makers adopt measures with the potential to influence medicines in LMICs, despite little evidence of their positive or negative effect. The EU's fragmented internal and external action related to pharmaceuticals also highlights the need for guiding EU principles for global equitable access to medicines, which in turn can support resilient global medicines supply chains and expand the pandemic preparedness and response efforts.

In addition to the global and EU-centric topics, two papers in the Series discuss the reforms and adaptations of health-care systems of Ireland, Bosnia and Herzegovina, and Croatia—given the uniqueness of their health systems (Ireland) and their experience of full-scale war in the past three decades (Bosnia and Herzegovina and Croatia). Currently, Ireland is the only state within the EU that does not provide universal health care, according to WHO criteria. The pandemic ensued in their third year of a 10-year plan for health reform that aimed to achieve universal health care in Ireland, Sláintecare. Sara Burke and colleagues analyse how the pandemic has accelerated Ireland's health system transformation and reform towards increasing resilience and delivering universal health care. Ana Marusic and colleagues evaluate how lessons from the wars have been applied to the pandemic 25 years later in Croatia (an EU member) and Bosnia and Herzegovina (not an EU member) and discuss newly recognised opportunities and improvements. The authors argue that health-care systems in Croatia and Bosnia and Herzegovina are not only resilient but antifragile, and that they benefited from stressors they were exposed to.

The COVID-19 pandemic has brought many ethical issues of health-care systems to public attention. Despite a substantial body of literature on ethical concerns in pandemics, these insights have not been broadly integrated into health system preparedness. Using the case of Germany as an example, Alena Buyx and colleagues reflect on four key ethical concerns related to the distribution of scarce resources, research ethics, structural inequities, and solidarity and social cohesion. Health systems need to proactively integrate ethical considerations into their design and operation, and ethicists need to be part of preparedness efforts from the beginning.

Aside from the COVID-19 pandemic, according to WHO, climate change might be the defining global public health threat of the 21st century. It has been unequivocally established that climate change is accelerating health security threat, with a 35% increase in rates of extreme weather events since the 1990s. The frequency, duration, and severity of extreme weather events in Europe are also projected to increase, including storms, heavy rainfall, floods, droughts, wildfires, or sea level rise, which can result in a sequence of events that lead to a succession of system failures. Jan Semenza and Shlomit Paz discuss climate change-related hazards, exposures, and vulnerabilities to infectious diseases, and describe observed impacts and projected risks, with policy entry points for adaptation to reduce these risks or avoid them altogether. Additionally, Jan Semenza discusses how European societies can tackle these inherent vulnerabilities to climate change by advancing lateral public health—a dendritic approach connecting government, and non-government bodies, with communities to enhance systemic resilience through participatory interventions, such as early warning systems, preparedness, and response. The approach hinges on community engagement in decision making by expanding social networks and social capital for climate change risk reduction and adaptation.

Although catastrophic events destabilise health systems, they also create opportunity for transformation. It is only a matter of time before the next health security threat emerges. It is also certain that building resilient health structures, both regionally and globally, will make the world better prepared for dealing with the next shock. Reforms need to be proactively integrated into the design and operation of health systems at the country level, as well as globally.

*The Lancet Regional Health – Europe*

